# Seasonal trends in surgical site infections after hernia repair

**DOI:** 10.1007/s10029-026-03668-w

**Published:** 2026-04-28

**Authors:** Janavi Sethurathnam, Chen Chia Wang, John Ewing, Aimal Khan, Joel F. Bradley

**Affiliations:** 1 Vanderbilt University School of Medicine, Nashville, TN USA; 2 Section of Surgical Sciences, Vanderbilt University Medical Center, Nashville, TN USA

**Keywords:** Hernia repair, Surgical site infection, NSQIP database, Seasonal trends

## Abstract

**Purpose:**

Surgical site infections (SSIs) after hernia repair are associated with longer hospital stays, increased readmission rates, and higher hospital costs. Patients undergoing colorectal and orthopedic surgeries during warmer months have been shown to have higher SSI risk. We investigated the relationship between season and SSI risk after hernia repair, hypothesizing that SSI risk is higher during warmer months.

**Methods:**

This retrospective cohort study used the American College of Surgeons (ACS) – National Surgical Quality Improvement Program (NSQIP) database to identify hernia repair patients from 2006 to 2021. We compared rates of any SSI between warm and cold seasons, defined based on admission quarter. Multi-variable and binomial logistic regression models were used to determine independent predictors of outcomes.

**Results:**

Of the 826,636 patients in the final cohort, 400,329 (48.4%) underwent surgery in the warm operative season. Warm operative season was associated with increased odds of superficial [OR 1.15 95% CI 1.10–1.20] (+ 1.11 SSIs per 1000 cases) and any [OR 1.12 95% CI 1.08–1.16] SSI (+ 1.30 SSIs per 1000 cases) after adjusting for covariates. There was no difference between seasons for rates of deep incisional [OR 1.04 95% CI 0.96–1.13] and organ space [OR 1.02 95% CI 0.93–1.12] SSIs. Other independent predictors of any SSI included open surgical approach, groin hernia, non-elective case type, smoking, diabetes, and obesity.

**Conclusions:**

Patients undergoing hernia repair in warmer months have a higher risk of superficial SSI compared to those in colder months. Season may represent an under-explored SSI risk factor and warrants further study to identify modifiable mechanisms.

**Supplementary Information:**

The online version contains supplementary material available at 10.1007/s10029-026-03668-w.

## Introduction

Surgical site infections (SSIs) are the leading cause of nosocomial infections in surgical patients and are associated with longer hospital stays, higher readmission rates, and higher hospital costs [1]. As one of the most common general surgical procedures in the USA, an estimated 611,000 ventral hernia repairs are performed annually. Therefore, mitigating SSIs after hernia repair surgery represents a significant population health and economic concern [2].

Reducing this burden requires an understanding of the risk factors for SSI after hernia repair. Well-established patient-related risk factors include obesity with a BMI > 35, current tobacco use, poorly controlled diabetes mellitus, and higher ASA classification [1, 3–6]. Procedure-related factors include surgical approach (open vs laparoscopic/robotic), mesh type and location, operative time, and increasing wound classification) [1, 4, 6]. Recent evidence has shown that surgery performed during warmer versus colder months is independently associated with increased risk of SSIs after colorectal (OR 1.04), spine (OR 1.15), orthopedic (OR 1.19), and plastic (OR 2.69) surgery [7–11]. Similar seasonal patterns have been observed after abdominopelvic and general surgery with summer having the highest SSI risk (OR 18.9 and OR 1.87) [12, 13]. Warmer and higher humidity conditions are theorized to cause increased growth of endogenous skin flora and drive SSIs [14, 15]. The relationship between operative season and risk of SSI after hernia repair remains unknown.

We believe that understanding seasonal SSI trends in hernia repair is important for SSI risk optimization and surgical planning. The goals of this study are (1) to investigate the relationship between warm versus cold operative season and the risk of SSIs after hernia repair and (2) to examine the temporal trends in rates of SSIs over the study period. We hypothesize that the risk of SSIs is higher during warmer months and that SSI rates have significantly declined from 2006–2021.

## Materials and methods

### Study design and data source

This was a retrospective cohort study conducted using the American College of Surgeons (ACS) – National Surgical Quality Improvement Program (NSQIP) database using data from 2006 to 2021. NSQIP collects de-identified information up to 30 days after surgery from patient records of its participating sites that include approximately 700 hospitals across the USA and globally, with majority of the sites located in Northern Hemisphere. This study was conducted according to the Strengthening the Reporting of Observational Studies in Epidemiology guidelines and was exempt from institutional review board approval due to the retrospective, publicly available, and deidentified nature of the database.

### Study population

We identified patients undergoing hernia repair (primary operation CPT codes: 49,560, 49,561, 49,565, 49,566, 49,570, 49,572, 49,585, 49,587, 49,590, 49,652, 49,653, 49,654.

**Fig. 1:**
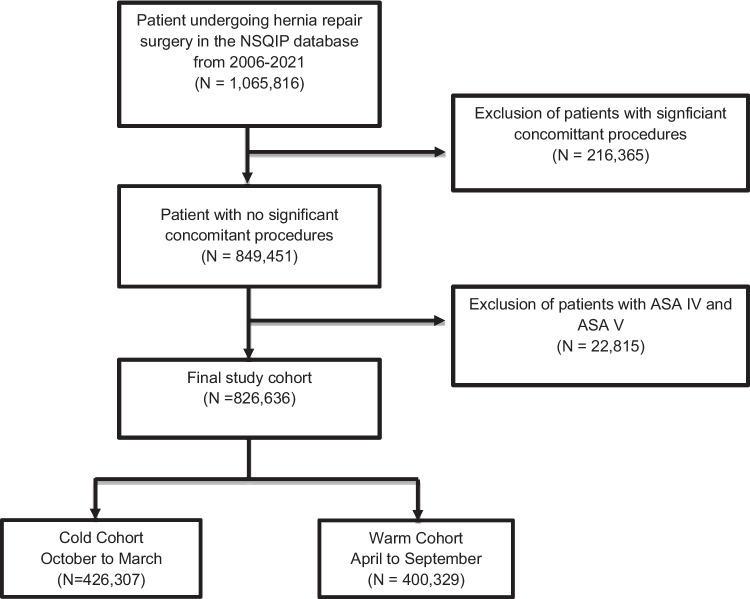
Patient selection flow diagram from the NSQIP database from 2006 to 2021 to our study patient population

49,655, 49,656, 49,657, 49,505, 49,507, 49,520, 49,521, 49,525, 49,550, 49,553, 49,555, 49,557, 49,650, 49,651) from 2006–2021 in the NSQIP database. Hernia types included initial and recurrent, reducible and incarcerated/strangulated, incisional, ventral, epigastric, umbilical, spigelian, femoral, and inguinal. These patients were further limited to those only undergoing additional procedures related to the hernia repair (additional procedure CPT codes: 49,568, 15,734, 15,830, 14,301, 14,302, 49,402). Those undergoing significant concurrent procedures and with ASA classes IV and V were excluded. Figure 1 is a complete study flow diagram.

### Outcomes and exposure

The primary outcome of this study was any SSI within 30 days of surgery. A patient was said to have any SSI if they had at least one superficial, deep, or organ space SSI. The primary exposure variable was operative season, defined based on the quarter in which a patient was admitted. Admission quarters were combined into warm (April to September) and cold (October to March) operative seasons. Secondary outcomes included superficial SSI, deep incisional SSI, organ space SSI, and the timing of SSIs (inpatient vs post-discharge). Other variables of interest included patient demographics, case type (elective vs non-elective), operative approach (open vs laparoscopic based on CPT codes), reducible vs incarcerated/strangulated hernia type (based on CPT codes), recurrence status of hernia (initial vs recurrent based on CPT codes), and operative duration.

### Statistical analysis

Continuous variables were represented as mean and standard deviation while categorical variables were represented as counts with percentages. Given the large sample size, the standard mean difference was calculated in addition to p-values. We used multi-variable logistic regression models to adjust for covariates and determine independent predictors of outcome SSI variables. We performed sub-group multi-variable regressions across clinically relevant strata. To improve clinical interpretability of our findings, we reported adjusted marginal predicted probabilities and adjusted absolute risk differences. To evaluate temporal and seasonal SSI trends, we used a binomial logistic regression. Patients with missing data for variables of interest were excluded from counts and regression models. All statistical analysis was done in R software version 4.5.1 (2025–06–13).

## Results

The cohort comprised 826,636 patients from the NSQIP database who underwent hernia repair from 2006–2021 without significant concurrent procedures and with ASA < IV. During the cold season (October–March), 426,307 (51.6%) of patients underwent surgery and 400,329 (48.4%) underwent surgery during the warm (April-September) season. In both cohorts, median age was 56 years and majority of patients were male (69% cold and 67% warm). Patient characteristics did not differ significantly between operative seasons (Table 1).

**Table 1:** Baseline characteristics of Cold and Warm Operative seasons

Variable	Cold *N* = 426,307 (51.6%)	Warm *N* = 400,329 (48.4%)
Age, mean (SD)	55.2 (15.5)	55.4 (15.9)
BMI, mean (SD)	30.1 (6.9)	30 (7.1)
Gender (%)		
Female	130,967 (30.7%)	130,538 (32.6%)
Male	295,134 (69.3%)	269,613 (67.4%)
Ethnicity (%) Hispanic	34,992 (9.3%)	34,552 (9.8%)
Non-Hispanic	339,629 (90.7%)	316,837 (90.2%)
Race (%)		
White	302,391 (70.9%)	280,477 (69.8%)
Black	38,386 (9.0%)	38,017 (9.5%)
Other	10,594 (2.5%)	10,667 (2.7%)
Unknown	74,936 (17.6%)	71,215 (17.8%)
Smoker (%)	75,027 (17.6%)	73,785 (18.4%)
Diabetes (%)	46,145 (10.8%)	44,545 (11.1%)
ASA Class (%)		
Class I	56,934 (13.4%)	52,466 (13.1%)
Class II	230,006 (54%)	212,413 (53.1%)
Class III	139,367 (32.7%)	135,450 (33.8%)
Case Type (%)		
Elective	397,138 (93.2%)	370,746 (92.6%)
Non-Elective	29,169 (6.8%)	29,582 (7.4%)
Hernia Type (%)		
Groin	174,130 (21.1%)	164,203 (19.9%)
Non-groin	252,177 (30.5%)	236,126 (28.6%)
Operative Approach (%)		
Laparoscopic	119,495 (28%)	111,102 (27.8%)
Open	306,812 (72%)	289,227 (72.2%)
Operative duration, minutes, mean (SD)	66.1 (48.7)	66.9 (49.6)

*SD* Standard deviation, *BMI* Body mass index; *ASA* American Society of Anesthesiologists.

In our unadjusted logistic regression analysis, the warm operative season was associated with increased odds of a superficial SSI [OR 1.17 95% CI 1.13–1.22], deep SSI [OR 1.10 95% 1.02–1.19], and any SSI [OR 1.15 95% CI 1.11–1.19] (Table 2). Superficial SSIs were the most prevalent type of SSI in both groups (1.03% and 1.21%), followed by deep incisional SSIs (0.28% and 0.30%), and organ space SSIs (0.22% and 0.23%) (Table 3). There was no difference in average hospital length of stay or the need for reoperation for SSIs between seasons. While not statistically significant, a greater proportion of SSIs occurred after discharge during warmer months.

**Table 2:** Unadjusted logistic regression for risk of surgical site infections by operative season (warm vs cold)

Variable	Unadjusted OR (95% CI)	*P*-value
Season – Warm (superficial SSI)	1.17 (1.13–1.22)	< 0.01
Season – Warm (deep incisional SSI)	1.10 (1.02–1.19)	0.018
Season – Warm (organ space SSI)	1.07 (0.98–1.17)	0.134
Season – Warm (any SSI)	1.15 (1.11–1.19)	< 0.01

**Table 3:** Post-operative outcomes of patients undergoing hernia repair stratified by operation during warm months (April to September) or cold months (October to March)

Variable	Cold *N* = 428,209 (51.6%)	Warm *N* = 402,090 (48.4%)	Standard Mean Difference
Any SSI (%)	6435 (1.51%)	6909 (1.73%)	0.017
Superficial SSI (%)	4393 (1.03%)	4845 (1.21%)	0.017
Deep Incisional SSI (%)	1177 (0.28%)	1208 (0.30%)	0.005
Organ Space SSI (%)	927 (0.22%)	928 (0.23%)	0.003
Any SSI prior to discharge (%)	609 (9.56%)	585 (8.53%)	−0.036
Length of stay, days, mean (sd)	0.77 (3.68)	0.79 (2.77)	0.007
Reoperation for incision and drainage (%)	835 (0.20%)	798 (0.20%)	0.001

*An SSI was defined as occurring prior to discharge when number of days from operation until SSI complication was less than length of stay

**Fig. 2:**
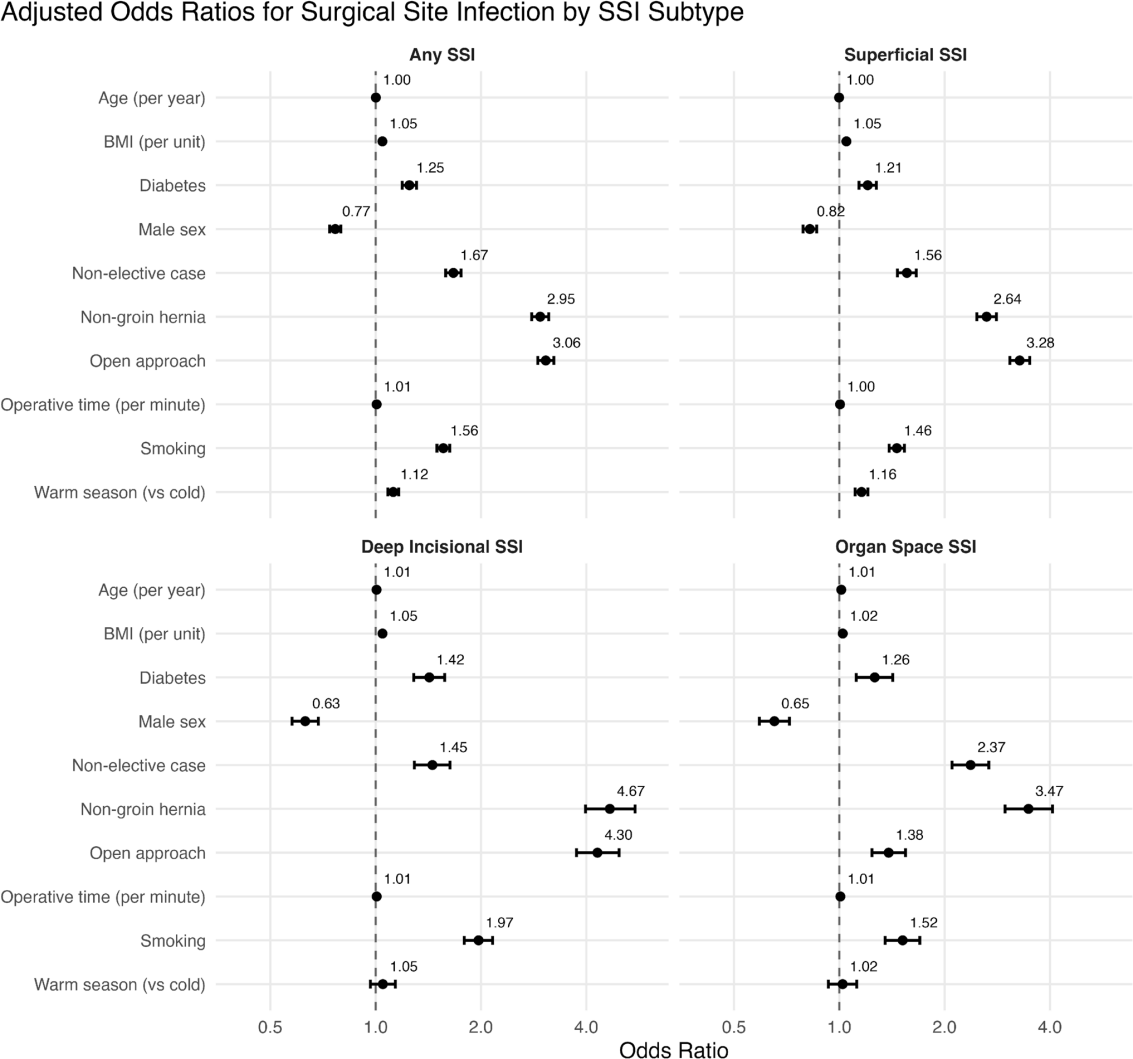
Multi-variable model for odds of surgical site infections after hernia repair

Warm operative season remained significantly associated with increased odds of superficial [OR 1.16 95% CI 1.11–1.21] and any [OR 1.12 95% CI 1.08–1.16] SSI after adjusting for operative approach, case type, smoking status, hernia type, co-morbid diabetes, BMI, operative duration, age, and sex (Fig. 2). Using marginal predicted probabilities, this translates to an additional 1.30 SSIs overall and 1.11 superficial SSIs per 1000 cases (Table 4). An open operative approach was the strongest predictor of superficial [OR 3.28 95% CI 3.07–3.50] and any SSI [OR 3.06 95% CI 2.91–3.23]. A non-groin hernia was the strongest predictor of deep incisional [OR 4.67 95% CI 3.98–5.51] and organ space [OR 3.47 95% CI 2.98–4.07] SSIs. Comparing the remaining predictors across SSI types (superficial, deep incisional, organ space), a non-elective case type [**OR 1.55** 95% CI 1.47–1.66 vs **OR 1.45** 95% CI 1.29–1.63 vs **OR 2.37** 95% CI 2.10–2.67], smoking [**OR 1.46** 95% CI 1.39–1.53 vs **OR 1.97** 95% CI 1.79–2.16 vs **OR 1.52** 95% CI 1.35–1.70], diabetes [**OR 1.21** 95% CI 1.14–1.28 vs **OR 1.42** 95% CI 1.28–1.58 vs **OR 1.26** 95% CI 1.12–1.42], and BMI [**OR 1.05** 95% CI 1.05–1.05 vs **OR 1.05** 95% CI 1.04–1.05 vs **OR 1.05** 95% CI 1.04–1.05] were all associated with increased odds of superficial, deep, and organ space SSIs, although the degree of increase varied between SSI types. Male sex was associated with a lower odds of all three types of SSIs [**OR 0.82** 95% CI 0.79–0.86 vs **OR 0.63** 95% CI 0.58–0.69 vs **OR 0.65** 95% CI 0.59–0.72]. Operative duration was associated with a higher risk of both deep [**OR 1.01** 95% CI 1.01–1.01] and organ space [**OR 1.01** 95% CI 1.01–1.01] SSIs while age was associated with a higher risk of only organ space [**OR 1.01** 95% CI 1.01–1.02] SSIs.

**Table 4:** Adjusted odds ratios, marginal predicted probabilities and risk differences for SSI subtypes by operative season

Outcome	Adjusted OR (95% CI)	Adjusted risk, Cold (95% CI)	Adjusted risk, Warm (95% CI)	Risk difference (per 1,000 cases) (95% CI)
Any SSI	1.12 (1.08–1.16)	1.07 (1.02–1.12)	1.20 (1.14–1.25)	1.30 (0.90 to 1.69)
Superficial SSI	1.16 (1.11–1.21)	0.71 (0.67–0.75)	0.82 (0.78–0.87)	1.11 (0.79 to 1.44)
Deep incisional SSI	1.05 (0.97–1.14)	0.14 (0.12–0.16)	0.15 (0.13–0.17)	0.07 (− 0.05 to 0.19)
Organ space SSI	1.02 (0.93–1.12)	0.21 (0.19–0.24)	0.22 (0.19–0.24)	0.05 (− 0.15 to 0.25)

Adjusted risks are marginal predicted probabilities from multivariable logistic regression, averaged over the covariates (age, sex, BMI, diabetes, smoking, case type, operative approach, operative time, and hernia type) in the study population.

Sub-group multivariable logistic regression analysis showed that the warm operative season was associated with an increased risk of any SSI across different clinically relevant strata. Hernia location (groin vs non-groin), case type (elective vs non-elective surgery), and operative approach (open vs laparoscopic) significantly impact risk of SSI. The warm operative season was associated with a higher odds of any SSI in the groin hernia group [OR 1.23 95% CI 1.11–1.35] compared to the non-groin hernia group [OR1.11 95% CI 1.07–1.15] (supplemental Table 1). Only elective hernia repairs had a significantly higher odds of any SSI in the warm season [OR 1.13 95% CI 1.09–1.17] compared to non-elective hernia repair [OR 1.08 95% CI 0.98–1.18] (supplemental Table 2). In both open and laparoscopic sub-groups, the warm season was associated with the same risk of any SSI [OR 1.12 95% 1.08–1.17 and OR 1.12 95% 1.01–1.23 respectively] (supplemental Table 3).

**Fig. 3:**
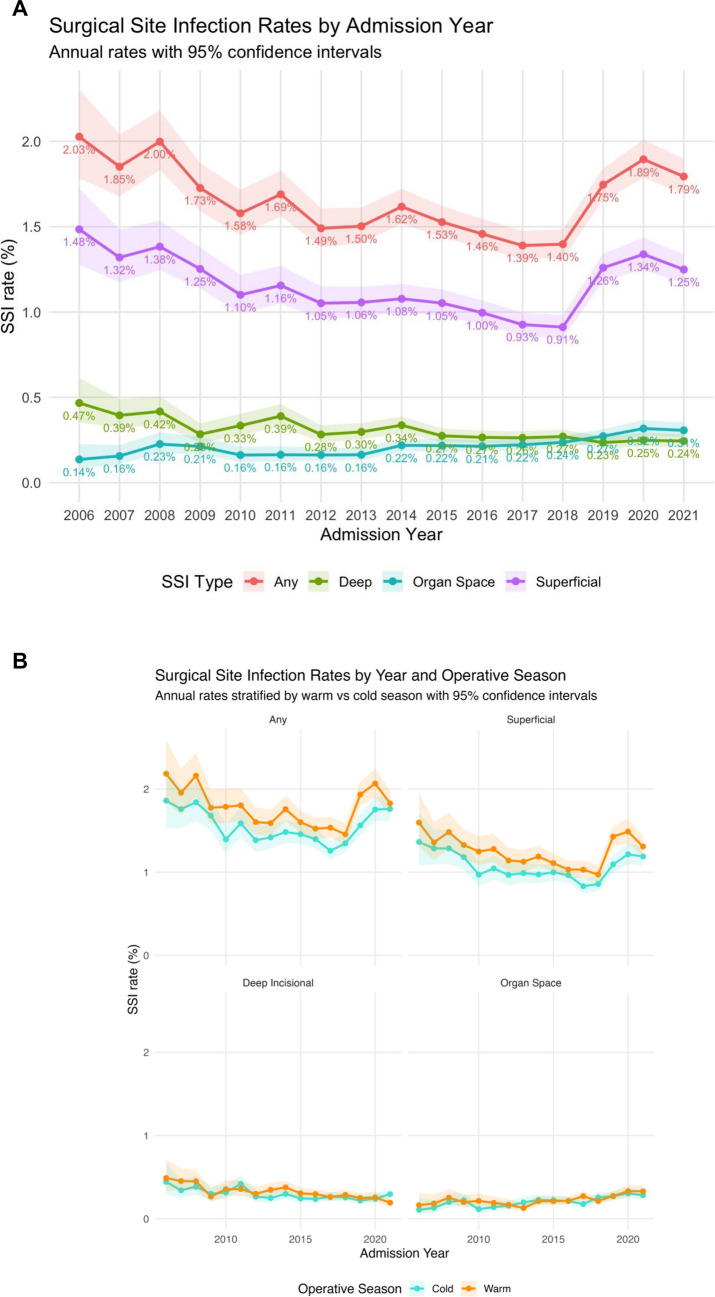
**A.** Surgical site infection rates across the study period (2006-2021) **B.** Surgical site infection rates in warm and cold operative seasons across the study period (2006-2021)

Temporal SSIs trends are demonstrated in Figs. 3A and 3B. Annual rates of any SSI remained relatively stable across the study ranging from 1.3–1.8% in the cold season and 1.5–2.2% in the warm season (Fig. 3B). In an unadjusted regression model with calendar year as a predictor of an SSI outcome, there was no significant change in risk of any SSI [OR 1.00 95% CI 0.99–1.00] or superficial SSI [OR 1.00 95% CI 0.99–1.00]. In contrast, calendar year was associated with lower odds of deep incisional SSI [OR 0.96 95% CI 0.96–0.97] and organ space SSI [OR 1.05 95% CI 1.04–1.06]. After adjusting for operative season, the findings remained the same: calendar year was not associated with change in risk of any SSI [OR 1.00 95% CI 0.99–1.00] or superficial SSI [OR 1.00 95% CI 0.99–1.00].

## Discussion

The results of this retrospective cohort study show that the warm operative season is independently associated with a greater risk of SSI after hernia repair surgery. This relationship is mostly driven by an increase in superficial SSIs during warmer seasons, with comparable rates of deep and organ space SSIs in warm vs cold seasons. This relationship held true across different clinical subgroups determined based on hernia type, case type, and operative approach. The proportion of SSIs occurring after hospital discharge did not differ between the warm and cold seasons. Other independent predictors of SSIs included a higher BMI, an open surgical approach, smoking, a non-elective case type, a groin hernia, and diabetes. SSI rates remained stable across the overall study periods.

Studies in spine, colorectal, and other general surgery patients have consistently demonstrated higher odds of SSIs in the summer months [8, 9, 11, 13]. Our results suggest that this pattern also applies to hernia repair patients. From the perspective of clinical relevance, effect sizes of our findings are low with the warm season only being associated with about 1 additional superficial or any SSI per 1000 cases. However, this increase is consistent and independent after adjustment for clinically relevant covariates. Given the volume of hernia operations, this absolute increase can translate to a meaningful number of additional infections at the population level. Unlike other season-based studies, we found that SSIs did not occur more frequently after discharge in summer months relative to winter months [7, 8].

Warm operative season was consistently associated with increased risk of any SSI across subgroups supporting that our primary findings are not driven by the overrepresentation of one subgroup. Our choice of sub-groups was determined by the combination of clinical relevance and variable availability in the NSQIP database. Groin hernia repairs, emergency surgery, and open surgery are all known to have higher SSI risks [1, 4, 6]. Consistency in our findings across subgroups offers additional support for our inclusion of the full spectrum of hernia repairs to evaluate the association between operative season and SSI risk at the population level.

While we observed a modest improvement in SSI rates over the 15-year period of our study, in absolute terms, the rates of SSIs were relatively stable. From 2006–2021, there have been more widespread adoption of enhanced recovery protocols, improvements in surgical technique, and increased use of minimally invasive approaches. SSI prevention care bundles developed based on CDC, ERAS, and WHO recommendations have been demonstrated to significantly reduce SSI burden after certain types of surgery [16]. These strategies include perioperative normothermia, perioperative normoglycemia, standardized antibiotic prophylaxis, chlorhexidine skin preparation, avoidance of hair shaving, and MRSA screening and decolonization [1, 17, 18]. However, given the persistence of SSI rates despite these measures, some experts suggest that we may be reaching the ceiling of SSI minimization with the above techniques [18]. The next step in SSI minimization may be to further examine the endogenous, patient-specific microbiological risk factors for SSI and consider how these factors might be addressed. The findings of our study, suggest previously unexamined exogenous factors like the operative season might be a worthwhile path to purse for further research. One hypothesis is that warmer temperatures could affect patient’s skin flora to change SSI risk. A better understanding of seasonal variability in skin microbiome in might better elucidate the mechanism behind the trends that we have observed and suggest whether different perioperative strategies (i.e. different types of skin prep) would be useful in warmer climates [19].

There are inherent limitations to using a large a national database like NSQIP. The large sample size renders even small differences between study groups statistically significant. To highlight when these differences are or are not clinically significant, we reported standard mean differences. While we sought to adequately power and control for confounding variables with our regression analysis, it is possible that unexamined or non-reported confounding factors may have affected SSI risk. For example, the type of mesh, defect size, and other operative factors may affect SSI risk but are not included in NSQIP [1, 3]. Despite the standardized NSQIP data reporting forms, there are likely variability in reporting standards and data collection methods between sites. Only patients with 30 days of post-operative data are included, which may exclude an important patient population who are lost to follow-up for systemic or other particular reasons. Perhaps most pertinent to our study, the lack of exact admission dates and hospital locations limits our ability to adjust for geographic variation in temperature between seasons. Unable to control for these geographic climate differences within the same month or season, we opted to assume that the average ambient temperature from October–March is cooler than that from April-September. The database includes countries with different geographic relationships to the equator; however, the predominance of US hospitals in this dataset allows us to reasonably examine overall national seasonal SSI trends. Further, due to the lack of exact hospital admission dates, we were unable to determine the hottest and coldest relied on calendar quarters to determine warm vs cold seasons. However, that we note an association between operative season and SSI risk despite this geographic heterogeneity, offers compelling evidence for further location- and month-adjusted analysis.

In summary, the warm operative season increases the odds of SSI after hernia repair compared to the cold season independent of other risk factors. Our study suggests a need to better explore this seasonal risk and the modifiable factors it might affect. Future research should examine the correlation between temperature changes on a regional level to SSI rate and investigate the relationship between climate, skin microbiome, and SSI. Ultimately, we are hopeful this could be translated into actionable practice changes.

## Supplementary Information

Below is the link to the electronic supplementary material.

ESM 1 (19.2 KB DOCX)

## References

[1] Tubre DJ, Schroeder AD, Estes J, Eisenga J, Fitzgibbons RJ (2018) Surgical site infection: the “Achilles heel” of all types of abdominal wall hernia reconstruction. Hernia 22(6):1003–1013. 10.1007/s10029-018-1826-930276561 10.1007/s10029-018-1826-9

[2] Schlosser KA, Renshaw SM, Tamer RM, Strassels SA, Poulose BK (2023) Ventral hernia repair: an increasing burden affecting abdominal core health. Hernia 27(2):415–421. 10.1007/s10029-022-02707-636571666 10.1007/s10029-022-02707-6

[3] Wilson RB, Farooque Y (2023) Correction to: Risks and prevention of surgical site infection after hernia mesh repair and the predictive utility of ACS-NSQIP. J Gastrointest Surg 27(1):211. 10.1007/s11605-022-05550-336471192 10.1007/s11605-022-05550-3PMC9877078

[4] Karamanos E, Kandagatla P, Watson J, Schmoekel N, Siddiqui A (2017) Development and validation of a scoring system to predict surgical site infection after ventral hernia repair: a Michigan Surgical Quality Collaborative study. World J Surg 41(4):914–918. 10.1007/s00268-016-3835-027872976 10.1007/s00268-016-3835-0

[5] Sereysky J, Parsikia A, Stone ME, Castaldi M, McNelis J (2020) Predictive factors for the development of surgical site infection in adults undergoing initial open inguinal hernia repair. Hernia 24(1):173–178. 10.1007/s10029-019-02050-331552553 10.1007/s10029-019-02050-3

[6] Kaoutzanis C, Leichtle SW, Mouawad NJ et al (2015) Risk factors for postoperative wound infections and prolonged hospitalization after ventral/incisional hernia repair. Hernia 19(1):113–123. 10.1007/s10029-013-1155-y24030572 10.1007/s10029-013-1155-y

[7] Anioke T, Fei Y, Stuart CM et al (2025) Seasonality of surgical site infection rates across a broad surgical sample and diverse health system. Am J Infect Control 53(5):559–564. 10.1016/j.ajic.2025.01.01839892855 10.1016/j.ajic.2025.01.018

[8] Wang CC, Sun K, Lee H et al (2025) Too hot to handle: investigating seasonal variations in surgical site infections after colorectal surgery. Surg Infect 26(6):405–412. 10.1089/sur.2024.29810.1089/sur.2024.29840008993

[9] Shuman WH, Baron RB, Gal JS et al (2022) Seasonal effects on surgical site infections following spine surgery. World Neurosurg 161:e174–e182. 10.1016/j.wneu.2022.01.10035093573 10.1016/j.wneu.2022.01.100

[10] Ogawa T, Yoshii T, Morishita S et al (2021) Seasonal impact on surgical site infections in hip fracture surgery: analysis of 330,803 cases using a nationwide inpatient database. Injury 52(4):898–904. 10.1016/j.injury.2020.10.05833082026 10.1016/j.injury.2020.10.058

[11] Duscher D, Kiesl D, Aitzetmüller MM et al (2018) Seasonal impact on surgical-site infections in body contouring surgery: a retrospective cohort study of 602 patients over a period of 6 years. Plast Reconstr Surg 142(3):653–660. 10.1097/PRS.000000000000467729878996 10.1097/PRS.0000000000004677

[12] Zhang J, Xue F, Liu SD et al (2023) Risk factors and prediction model for inpatient surgical site infection after elective abdominal surgery. World J Gastrointest Surg 15(3):387–397. 10.4240/wjgs.v15.i3.38737032800 10.4240/wjgs.v15.i3.387PMC10080607

[13] Ichida K, Noda H, Maemoto R et al (2024) Contrasting seasonality of the incidence of incisional surgical site infection after general and gastroenterological surgery: an analysis of 8436 patients in a single institute. J Hosp Infect 151:140–147. 10.1016/j.jhin.2024.06.00338950864 10.1016/j.jhin.2024.06.003

[14] Anthony CA, Peterson RA, Polgreen LA, Sewell DK, Polgreen PM (2017) The seasonal variability in surgical site infections and the association with warmer weather: a population-based investigation. Infect Control Hosp Epidemiol 38(7):809–816. 10.1017/ice.2017.8428506327 10.1017/ice.2017.84PMC5832937

[15] Liou RJ, Earley MJ, Forrester JD (2022) Effect of climate on surgical site infections and anticipated increases in the United States. Sci Rep 12(1):19698. 10.1038/s41598-022-24255-w36385136 10.1038/s41598-022-24255-wPMC9668825

[16] Zywot A, Lau CSM, Stephen Fletcher H, Paul S (2017) Bundles prevent surgical site infections after colorectal surgery: meta-analysis and systematic review. J Gastrointest Surg 21(11):1915–1930. 10.1007/s11605-017-3465-328620749 10.1007/s11605-017-3465-3

[17] Cunha T, Miguel S, Maciel J, Zagalo C, Alves P (2025) Surgical site infection prevention care bundles in colorectal surgery: a scoping review. J Hosp Infect 155:221–230. 10.1016/j.jhin.2024.10.01039486458 10.1016/j.jhin.2024.10.010

[18] Seidelman JL, Mantyh CR, Anderson DJ (2023) Surgical site infection prevention: a review. JAMA 329(3):244–252. 10.1001/jama.2022.2407536648463 10.1001/jama.2022.24075

[19] Townsend EC, Xu K, De La Cruz K et al (2025) Still not sterile: viability-based assessment of the skin microbiome following pre-surgical application of a broad-spectrum antiseptic reveals transient pathogen enrichment and long-term recovery. Microbiol Spectr 13(5):e0287324. 10.1128/spectrum.02873-2440207941 10.1128/spectrum.02873-24PMC12054058

