# Supplementary material for: Seasonal trends in surgical site infections after hernia repair
Source: Hernia. 2026 Apr 28;30(1):187. doi: 10.1007/s10029-026-03668-w (PMC13124950; doi:10.1007/s10029-026-03668-w)
Supplement: Supplementary file 1 — (19.2 KB DOCX) [file 10029_2026_3668_MOESM1_ESM.docx]

Supplemental Information

Supplemental Table 1. Hernia type by operative approach

| Hernia Type | Count  N=826,636 (%) |
| --- | --- |
| Open |  |
| Non-Groin | 360,426 (43.6) |
| Groin | 235,613 (28.5) |
| Laparoscopic |  |
| Non-Groin | 127,877 (15.5) |
| Groin | 102,720 (12.4) |
| *Non-Groin hernias: ventral, incisional, epigastric, umbilical, and spigelian*  *Groin hernias: femoral and inguinal* | |

Supplemental Table 2. Multivariable logistic regression subgroup analysis: adjusted odds of any surgical site infection after groin and non-groin hernia repair

|  | Groin Hernia Subgroup | | Non-Groin Hernia Subgroup | |
| --- | --- | --- | --- | --- |
| Variable | **OR (95% CI)** | **p-value** | **OR (95% CI)** | **p-value** |
| Warm season (vs cold) | **1.23 (1.11–1.35)** | **<0.001** | **1.11 (1.07–1.15)** | **<0.001** |
| Age (per year) | 1.00 (1.00–1.00) | 0.252 | 1.00 (1.00–1.00) | 0.016 |
| Male sex | 0.52 (0.46–0.59) | <0.001 | 0.79 (0.76–0.82) | <0.001 |
| BMI (per unit) | 1.06 (1.05–1.07) | <0.001 | 1.04 (1.04–1.05) | <0.001 |
| Diabetes | 1.25 (1.05–1.47) | 0.010 | 1.25 (1.19–1.31) | <0.001 |
| Smoking | 1.56 (1.39–1.75) | <0.001 | 1.56 (1.50–1.64) | <0.001 |
| Non-elective case | 2.54 (2.20–2.91) | <0.001 | 1.58 (1.50–1.67) | <0.001 |
| Open approach | 1.88 (1.66–2.14) | <0.001 | 3.32 (3.13–3.52) | <0.001 |
| Operative time (per minute) | 1.00 (1.00–1.00) | <0.001 | 1.01 (1.01–1.01) | <0.001 |

Supplemental Table 3. Multivariable logistic regression subgroup analysis: adjusted odds of any surgical site infection after elective and non-elective hernia repair

|  | Elective Repair | | Non-Elective Repair | |
| --- | --- | --- | --- | --- |
| Variable | **OR (95% CI)** | **p-value** | **OR (95% CI)** | **p-value** |
| Warm season (vs cold) | **1.13 (1.09–1.17)** | **<0.001** | **1.08 (0.98–1.18)** | **0.110** |
| Age (per year) | 1.00 (1.00–1.00) | 0.090 | 1.00 (1.00–1.00) | 0.602 |
| Male sex | 0.76 (0.73–0.79) | <0.001 | 0.84 (0.76–0.92) | <0.001 |
| BMI (per unit) | 1.05 (1.05–1.05) | <0.001 | 1.03 (1.03–1.04) | <0.001 |
| Diabetes | 1.25 (1.19–1.32) | <0.001 | 1.24 (1.11–1.39) | <0.001 |
| Smoking | 1.58 (1.51–1.65) | <0.001 | 1.40 (1.26–1.56) | <0.001 |
| Non-groin hernia | 3.02 (2.84–3.21) | <0.001 | 2.39 (2.07–2.77) | <0.001 |
| Open approach | 3.11 (2.95–3.29) | <0.001 | 2.73 (2.33–3.21) | <0.001 |
| Operative time (per minute) | 1.01 (1.01–1.01) | <0.001 | 1.00 (1.00–1.01) | <0.001 |

Supplemental Table 3. Multivariable logistic regression subgroup analysis: adjusted odds of any surgical site infection after open and laparoscopic hernia repair

|  | Open Repair | | Laparoscopic Repair | |
| --- | --- | --- | --- | --- |
| Variable | **OR (95% CI)** | **p-value** | **OR (95% CI)** | **p-value** |
| Warm season (vs cold) | **1.12 (1.08–1.17)** | **<0.001** | **1.12 (1.01–1.23)** | **0.025** |
| Age (per year) | 1.00 (1.00–1.00) | 0.034 | 1.00 (1.00–1.00) | 0.873 |
| Male sex | 0.76 (0.73–0.79) | <0.001 | 0.79 (0.71–0.88) | <0.001 |
| BMI (per unit) | 1.05 (1.04–1.05) | <0.001 | 1.04 (1.03–1.04) | <0.001 |
| Diabetes | 1.25 (1.19–1.31) | <0.001 | 1.23 (1.08–1.40) | 0.001 |
| Smoking | 1.60 (1.53–1.68) | <0.001 | 1.30 (1.15–1.47) | <0.001 |
| Non-groin hernia | 3.14 (2.95–3.34) | <0.001 | 2.19 (1.90–2.53) | <0.001 |
| Non-elective case | 1.64 (1.56–1.73) | <0.001 | 1.89 (1.60–2.22) | <0.001 |
| Operative time (per minute) | 1.01 (1.01–1.01) | <0.001 | 1.01 (1.00–1.01) | <0.001 |
